# Iron and Zinc Foliar Spraying Affected *Sideritis cypria* Post. Growth, Mineral Content and Antioxidant Properties

**DOI:** 10.3390/plants14060840

**Published:** 2025-03-07

**Authors:** Antonios Chrysargyris, Nikolaos Tzortzakis

**Affiliations:** Department of Agricultural Sciences, Biotechnology and Food Science, Cyprus University of Technology, 3603 Limassol, Cyprus; a.chrysargyris@cut.ac.cy

**Keywords:** microelements, hydroponics, antioxidants, *Sideritis* spp., biofortification

## Abstract

Species of the genus *Sideritis* are gaining heightened recognition for their applications in both culinary and industrial contexts. The improvement of crop cultivation techniques to promote the quality of the final product is imperative nowadays for ensuring sustainable and successive agricultural production, especially for medicinal and aromatic plant species. The present study examined the impacts of foliar application of iron (Fe) and zinc (Zn) on *Sideritis cypria* plants grown in hydroponics. The spraying of Fe (1.79 mM and 10.79 mM) and Zn (1.74 mM and 10.43 mM Zn) was applied four times at 10-day intervals, and the effects on plant growth, plant physiology, antioxidant status and nutrient uptake were investigated. The applications of both the high Fe and Zn levels decreased the plant yield and dry matter content. The use of the high Fe levels, particularly, resulted in elevated oxidative stress, as indicated by the increased levels of lipid peroxidation and hydrogen peroxide production and the increased peroxidase enzymatic activity. The application of the high Fe levels (10.79 mM Fe) also induced the plants’ non-enzymatic antioxidant mechanisms and the total flavonoid content. All foliar applications increased the accumulation of sodium in the leaf tissue. The plants’ calcium content was increased after the treatment with Zn, while the magnesium content was increased only when the high Zn level (10.43 mM Zn) was applied. Interestingly, the foliar application of both Zn and Fe had no effect on the build-up of zinc or iron content in the leaf tissue. Biofortification with minerals is a key approach to enhancing the biological quality and the nutritional value of plants, while its foliar application or application via different fertigation strategies needs to be evaluated either as single or as combined practices.

## 1. Introduction

There is an increasing fascination with studying unexplored native medicinal and aromatic plants (MAPs) and their qualities [1,2]. *Sideritis* spp. are one of these genera that have a significant global economic impact [3]. They are predominantly represented by annual or perennial aromatic plants (or shrubs) that exist in the tropical and subtropical regions of the Northern hemisphere, primarily in the Mediterranean basin [4]. *Sideritis* essential oils (EOs) are employed as lung-disinfecting agents, diuretics, stomachics and neurorelaxants. *S. cypria* is an indigenous species of flora in Cyprus (https://www.flora-of-cyprus.eu, assessed on 27 December 2024). The first description of the chemical profile of the *S. cypria* EO was conducted on native populations harvested in the northern part of Cyprus during the flowering period [5]. In fact, various plant-growing conditions (such as reduced irrigation practices, organic rather than conventional fertigation, successive samplings) were evaluated. The findings suggested that cultivation practices alter the plant physiology and the chemical characteristics of the EOs by accelerating or delaying the plants’ metabolic processes [6]. Recent research has examined the effects of environmental factors (e.g., varying altitudes, mountainous vs. plain regions) and seasonal variables on antioxidant capacity and EO profiles [7]. The EO profile of the species is dominated by *β*-phellandrene, *β*-pinene and *α*-pinene, with seasonal variations indicating a substantial link between altitude and season. However, *Sideritis* is characterized as an oil-poor genus and is less valued for its EO yield and use in the cosmetics and perfume industry. In contrast, *Sideritis* infusions have a pleasant aroma, are used as herbal teas and are recognized for their antibacterial and antifungal properties [5].

The growing interest in investigating MAPs and their qualities has led to an increased industry demand for consistent and high-quality MAP products. The lack of growers’ knowledge and practical horticultural methods highlights the need for research on sustainable cultivation and fertilization techniques for MAPs as well as their impact on environmental and human health [8]. For under-exploited species like *S. cypria*, there is a lack of information regarding crop requirements for sustainable farming. Only very recently, research has been conducted on tailoring nutrient applications to enhance *S. cypria* plant growth and stimulate its secondary metabolism [9]. In addition to adjusting mineral intake and demands through the root absorption in cultivation, foliar mineral treatment is also applied [10], particularly with micronutrients, to alleviate nutrient deficiencies or oxidative stress caused by various abiotic and biotic factors [11,12,13]. Moreover, foliar application of micronutrients may enhance their accumulation in plant tissues, thereby increasing their levels and/or influencing the balance of other minerals. Mineral deficiencies significantly impact human health, with iron (Fe) and zinc (Zn) deficiencies being major concerns in developing countries. These deficiencies contribute to increased mortality rates, greater susceptibility to infections and anemia [14] and a decline in the Gross Domestic Products (GDPs) of developing nations by 2–5% [15,16].

Effective biofortification is critical, and foliar fertilization must address both mineral delivery to edible plant parts and their penetration. The efficiency of foliar treatment varies depending on exogenous and environmental factors such as light, temperature, wind, the time of application, photoperiod, air humidity, any relevant plant stress and the mineral composition used [17]. Among the endogenous factors, the thickness of the cuticle covering the epidermal cells, the number of cuticular pores and the leaf location also play significant roles [17]. It has been reported that the lower leaf surface, which has a higher density of stomata, may absorb more Fe components compared to the upper leaf surface [18].

Iron and Zn are both required microelements for plant growth and have important roles in a variety of metabolic processes, either as metal components of enzymes or as functional, structural or regulatory cofactors [19,20]. Previous studies have investigated the impacts of micronutrient foliar applications on plant development and EO production of MAPs, including chamomile (*Matricaria chamomilla* L.) [21], sweet basil (*Ocimum basilicum* L.) [22,23], summer savory (*Satureja hortensis* L.) [24], spearmint (*Mentha spicata* L.) [20], lavender (*Lavandula angustifolia* Mill. [25], lemon balm (*Melissa officinallis* L.) [26,27], coriander (*Coriandrum sativum* L.) [28], rosemary (*Rosmarinus officinalis* L.) [29], dragonhead plants (*Dracocephalum moldavica* L.) [19,30], borago (*Borago officinalis* L.) [31], purslane (*Portulaca oleracea* L.) [32], peppermint (*Mentha piperita* L.) [12,33] and marigold (*Calendula officinalis* L.) [31]. More recent studies on a series of different plant species revealed the rising interest in foliar nutrient spraying as a strategy to boost crop yields and plant properties. The foliar application of minerals, including Zn and Fe, significantly increased growth parameters and antioxidant capacity in basil plants [34] and showed a significant positive impact on the yield, micronutrient concentration and uptake of Indian mustard *(Brassica juncea*) [35] and increased grain yield in rice [36]. Moreover, as reviewed by Ikram et al. [37], iron enrichment in tomato plants improved a series of quality parameters: total soluble content, antioxidant capacity, total flavonoid and phenolic content and total sugars.

The aim of this study was to evaluate the growth and physiological traits, antioxidant capacity and nutrient accumulation in *S. cypria* plants grown in hydroponics and subjected to foliar application of Fe and Zn and compare them with control plants that were sprayed with water.

## 2. Results and Discussion

**Figure 1:**
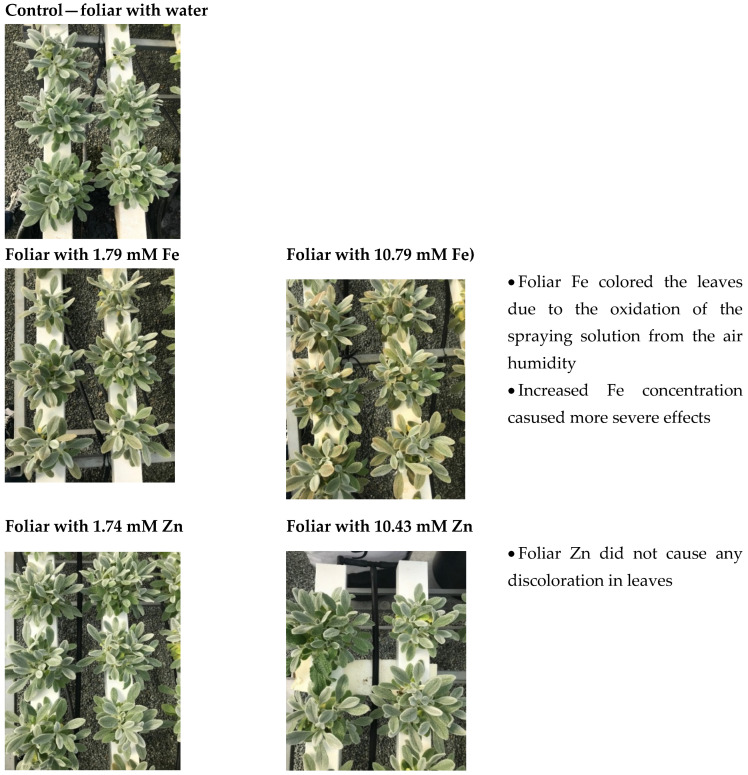
Illustration of *Sideritis cypria* plants grown in hydroponics after foliar applications (foliar with dH_2_O—control, 1.79 mM Fe, 10.79 mM Fe, 1.74 mM Zn and 10.43 mM Zn).

The effects of the Fe and Zn foliar spraying on the *S. cypria* biomass production are shown in Table 1. The foliar treatments of 10.79 mM Fe, 1.74 mM Zn and 10.43 mM Zn had negative impacts on the biomass fresh weight per plant when compared to the control treatment. Although there were no discernible effects on the biomass dry weight, these treatments raised the dry matter content of the plant biomass by up to 22.8% when compared to the control treatment. When compared with the plants that were not sprayed, the *Mentha piperita* fresh weight increased when the Fe was sprayed; however, the rise was more pronounced in the plants with lower Fe concentrations than those with higher concentrations [38]. Furthermore, previous reports have indicated that Zn and Fe foliar spraying significantly increased the yield of crops, including chamomile [21] and basil [23]. However, the above-mentioned species have quite thin leaves and structures compared with the thick leaves that *Sideritis* has (see Figure 1), and that could be a reason for the less-effectiveness of the spraying applications in the present study. Previous reports on amaranth (*Amaranthus* spp.) found no differences in plant fresh weight after the application of 100 ppm of ZnSO_4_ (which is 0.62 mM of Zn); however, the application of biosynthesized Zn nanoparticles (combining ZnSO_4_ with moringa—*Moringa oleifera* L.—leaf extract) increased the fresh weight compared with water-treated plants [39], and this provides new insights on combining materials for improving foliar application efficiency. The reason for the decrease in the plant growth parameters found in our study could be due to the long-term exposure of the plants to any sort of stress, the foliar applications in this case, that adversely affects the growth and development of plants [40].

The non-destructive testing revealed an increase in the relative chlorophyll content (SPAD) with the foliar spraying of 1.74 mM Zn, while the higher Zn levels induced reductions in the SPAD (Table 2). No significant effects were observed on the leaf chlorophyll fluorescence among the examined treatments, which was recorded as Fv/Fm above the 0.8 that is considered the critical value [41]. Specifically, chlorophyll fluorescence can reveal how resilient a plant is to environmental stressors and how much the photosynthetic system has been impacted by them [42]. Additionally, the plants sprayed with 1.74 mM Zn had the greatest levels of chlorophyll a and total chlorophylls, whereas the plants sprayed with 1.79 mM Fe had the lowest levels, as well as lower amounts of carotenoids. Previous research found that Fe and Zn foliar spraying on rosemary grown in perlite stimulated the chlorophyll content [29]; however, this was not evidenced in the present work, most probably due to the different cultivation practice, foliar doses applied, plant species and crop duration. Additionally, Ruiz-Torres et al. [43] reported that Zn application in soil in coriander (*Coriandrum sativum*) enhanced the chlorophyll content, and this can be related to the higher Zn doses used (up to 400 mg/kg), cultivation system (soil versus hydroponics) and plant species tested (coriander versus *Sideritis*) compared with the present study. The above provided insights that both Zn and Fe levels could be increased, if applied through the root system, by increased concentrations in the supplied NS. Hydroponics is an effective high-tech cultivation system to tailor mineral applications in the root for maximization of mineral efficiency; however, additional research is needed in that direction before any conclusions can be drawn. Additionally, being under mineral efficiency, hydroponically grown plants might not need any additions of minerals, and the excess of a mineral could disrupt the electron and energy transport in the photosystem. Therefore, moderate amounts of Fe or Zn are beneficial for improving the electron and energy transport properties of the photosystem, while spraying high concentrations of Fe negatively affects plant features [44]. Moreover, even though Fe is crucial for chlorophyll formation, foliar-applied Fe may hamper leaf penetration and movement in the apoplast because of the negatively charged plant cuticles and cell walls that cannot promote this feature [45]. Chlorophyll b levels did not change by Fe spraying in pepper plants [46], which is consistent with the current observations. Lastly, when 1.79 mM Fe and 10.79 mM Fe were applied, the carotenoids:total chlorophylls ratio was reduced in comparison to the control and 10.43 mM Zn treatments. Since the ratio of carotenoids to total chlorophylls is an indicator of vegetative photosynthesis, plant growth and stress responses [47], the higher MDA values suggest that the Fe spraying was stressing the plants more than the control plants. It has been demonstrated that under nutrient stress or senescence of leaves, carotenoids have been shown to decrease more slowly than chlorophylls, and this leads to deviation in their ratios [48], which has been shown to vary among plant species [49].

**Figure 2:**
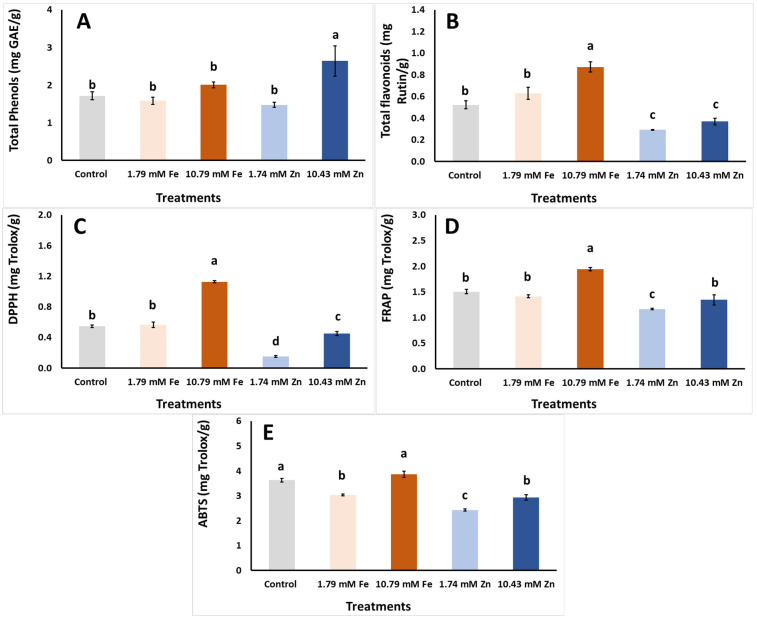
Effects of foliar applications (foliar with dH_2_O—control, 1.79 mM Fe, 10.79 mM Fe, 1.74 mM Zn and 10.43 mM Zn) on *Sideritis cypria* on (**A**) total phenols (mg gallic acid equivalents/g of fresh weight, mg GA/g FW); (**B**) flavonoids (mg rutin/g FW); and antioxidant activity as assayed by (**C**) 2,2-diphenyl-1-picrylhydrazyl (DPPH), (**D**) ferric reducing antioxidant power (FRAP) and (**E**) 2,2′-azino-bis(3-ethylbenzothiazoline-6-sulphonic acid (ABTS) (mg Trolox/g FW) in plants grown in hydroponics, with NPK values of 150-50-350 mg/L. Significant differences (*p* < 0.05) among foliar applications are represented by different letters.

The effects of the various foliar applications on the total phenolic compound content, total flavonoids and antioxidant capacity are presented in Figure 2. In the case of the total phenolics, a noticeable rise was revealed in the plants sprayed with 10.43 mM Zn over the other applications (Figure 2A). Furthermore, the antioxidant capacity (assayed by DPPH and FRAP) (Figure 2C,D), as well as the content, of the flavonoids (Figure 2B) of the *S. cypria* plants was increased with the foliar application of 10.79 mM Fe, while significant decreases were observed with both Zn foliar applications. Similarly, in the ABTS, decreases were observed with the applications of 1.79 mM Fe, 1.74 mM Zn and 10.43 mM Zn over the control and 10.79 mM Fe treatment (Figure 2E). The total flavonoid content was increased after the Fe and Zn spraying in the lemon balm (*Melissa officinallis* L) leaves, while the beneficial effects of the coupled microelements in the foliar spraying were demonstrated [50,51]. The increase in the total flavonoids in our study after spraying with high Fe levels can be connected to the fact that Fe binds with the key enzymes anthocyanin synthase and flavanone 3-hydroxylase, which are crucial in the biosynthesis pathways of flavonoids [52]. When Zn and Fe were applied to rosemary plants, their total flavonoid contents increased [29]. However, in the present study, this was seen only with the application of the high Fe spraying and not with the Zn spraying, with the latter reversing the flavonoid levels, causing significant decreases compared to the control. Indeed, the high-Zn foliar spray stimulated the production of the total phenols compared to the control, indicating potentially different mechanisms involved toward the antioxidant capacity of the examined species. No changes in total phenol content were observed in earlier research on spearmint sprayed with zinc at low doses, which is in line with the current findings [53]. Notably, the beneficial effects of Zn (including K and Si)-spraying applications were more profound in spearmint [53] and lavender [25] plants when subjected to salinity stress when the antioxidant mechanisms (both non-enzymatic and enzymatic) were activated. As for Fe, the enhanced antioxidant capacities (DPPH, FRAP, ABTS) as a result of the high Fe treatment suggest that foliar Fe application significantly alters the flavonoid metabolic profiles of *S. cypria* plants, which translates to corresponding increases in the non-enzymatic antioxidant mechanism.

**Figure 3:**
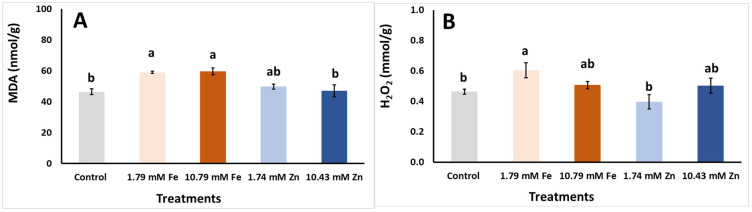

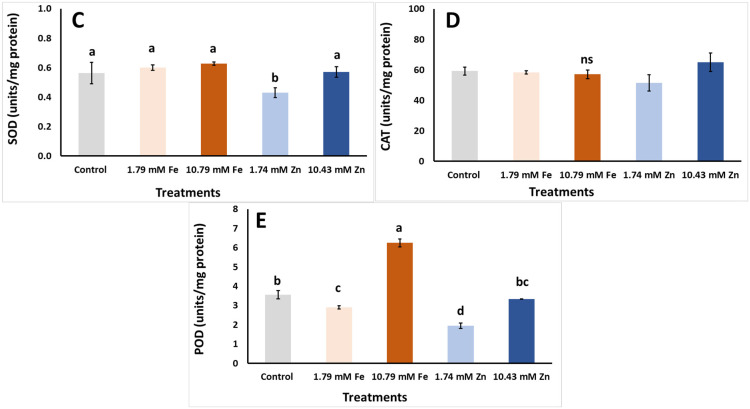
Effects of foliar applications (foliar with dH_2_O—control, 1.79 mM Fe, 10.79 mM Fe, 1.74 mM Zn and 10.43 mM Zn) on *Sideritis cypria* on (**A**) lipid peroxidation—MDA (nmol/g); (**B**) hydrogen peroxide—H_2_O_2_ (μmol/g); and enzymatic antioxidant activity of (**C**) superoxide dismutase—SOD (units/mg protein), (**D**) catalase—CAT (units/mg protein) and (**E**) peroxidase—POD (units/mg protein), in plants grown in hydroponics, with NPK values of 150-50-350 mg/L. Significant differences (*p* < 0.05) among foliar applications are represented by different letters. ns: no significant differences.

The different foliar applications affected the examined damage indicators (H_2_O_2_ and MDA) and enzymatic antioxidant activity (SOD, CAT and POD) of the *S. cypria* plants grown in hydroponics (Figure 3). The hydrogen peroxide production substantially increased with the foliar spraying of 1.79 mM Fe compared to the control and 1.74 mM Zn treatment (Figure 3B), while both Fe foliar applications induced an increase in MDA content when compared to the control and the 10.43 mM Zn (Figure 3A). Regarding the enzyme activity, the SOD activity was decreased with the foliar application of 1.74 mM Zn (Figure 3C), while the POD was reduced, with the application of both 1.74 mM Fe and 1.74 mM Zn, and increased, with the application of 10.79 mM Fe, over the control treatment (Figure 3E). No changes were shown to the CAT activity in the plants among the tested applications (Figure 3D). Previous reports have indicated that Zn in high concentrations may induce the generation of H_2_O_2_ and MDA (mirroring the lipid peroxidation status) and this might lead to a reduction in photosynthetic pigment biosynthesis, an effect that was revealed in our study as well [43,54]. However, in the present research, Zn spraying did not cause any stress in the *Sideritis* plants, primarily due to the relatively low concentrations used and the characteristics of the examined plant species. Indeed, Ruiz-Torres et al. [43] reported that Zn application in soil at high doses in coriander (*Coriandrum sativum*) induced oxidative stress to the plants, whereas in lower Zn doses, the plants were able to mitigate stress through the significant induction of several antioxidant enzymatic mechanisms, including CAT, POD and ascorbate peroxidase—APX. In the present research, this antioxidant enzyme stimulation was not evidenced; most probably, it was not initiated by any stress conditions after the Zn spraying.

**Figure 4:**
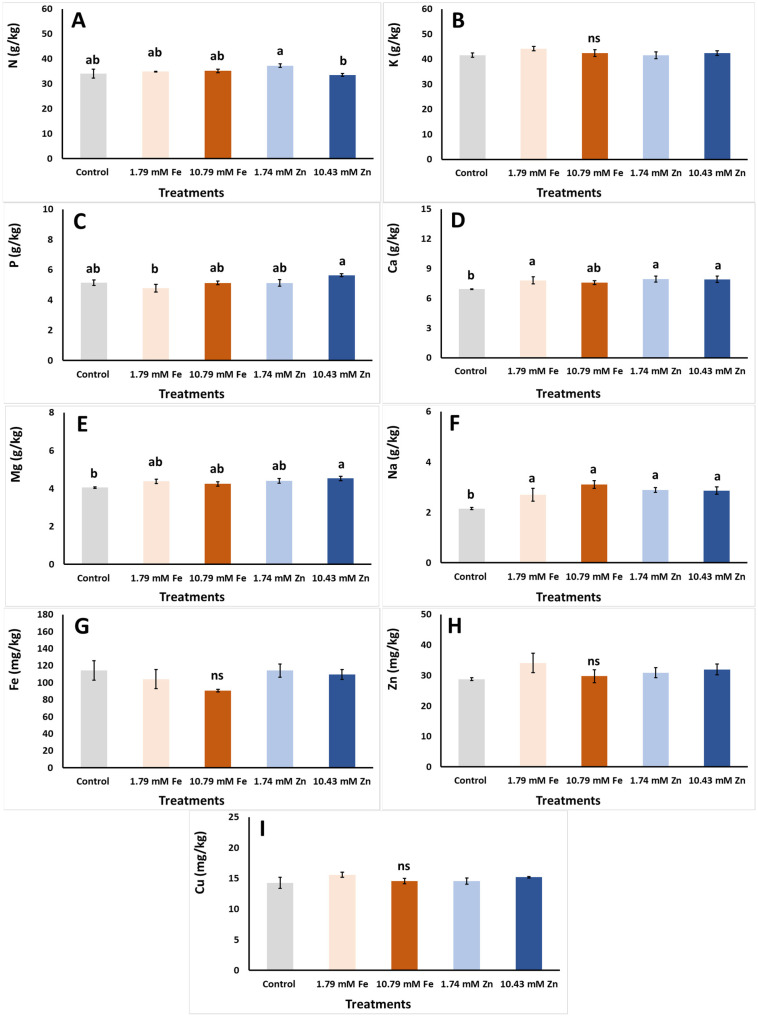
Effects of foliar applications (foliar with dH_2_O—control, 1.79 mM Fe, 10.79 mM Fe, 1.74 mM Zn and 10.43 mM Zn) on *Sideritis cypria* macronutrient [(**A**) nitrogen—N, (**B**) potassium—K, (**C**) phosphorus—P, (**D**) calcium—Ca, (**E**) magnesium—Mg and (**F**) sodium—Na] and micronutrient [(**G**) iron—Fe, (**H**) zinc—Zn and (**I**) copper—Cu] contents in plants grown in hydroponics, with NPK values of 150–50–350 mg/L. Significant differences (*p* < 0.05) among foliar applications are represented by different letters. ns: no significant differences.

The contents of the minerals accumulated in the *S. cypria* plants grown hydroponically, as affected by the different Fe and Zn foliar applications, are presented in Figure 4. The highest leaf N content was found in the plants that received the 1.74 mM Zn spraying, while the N was decreased with the application of 10.43 mM Zn (Figure 4A). This application, however, resulted in the plant production with the greatest P level, while a reduction occurred with the application of 1.79 mM Fe (Figure 4C). Overall increases in the Na, Ca and Mg levels in the plants occurred with the application of all foliar applications in comparison to the control application (Figure 4D–F), while, finally, no significant variations were found in the K (Figure 4B) and micronutrient contents (Fe, Zn and Cu) (Figure 4G–I) of the *S. cypria* plants.

The applications of nano-Zn and nano-Fe did not impact the K content of the rosemary [29], which is consistent with the results of the current research on the effects of Zn on K content but contradicts prior reports on Fe-sprayed pepper plants treated with inorganic Fe salts [46]. Previous investigations have suggested that foliar micronutrient administrations such as N and K [55], in conjunction with foliar macronutrient use or with an appropriate macronutrient fertilization scheme [56], may enhance the effective uptakes of Zn and Fe.

All micronutrient levels in the present research were within the typical range found in plant dry matter, as reported previously [17]. Specifically, the Fe content averaged 106.6 mg/kg, the Cu content averaged 14.8 mg/kg and the Zn content averaged 31.1 mg/kg, all of which fall within the common ranges for typical levels: Fe, 50–250 mg/kg; Cu, 5–20 mg/kg; and Zn, 21–150 mg/kg [17]. Foliar Fe application requires more time to enter plant leaf tissue than Zn, with an estimated absorption period of 10–20 days. This delay could also explain the lower effectiveness of the Fe application in increasing the Fe accumulation in the plant tissue [17]. The zinc application in the arugula (*Eruca sativa* L.) growing in soil did not affect the Cu and Fe content [57], which is consistent with the present observations. However, several authors have indicated that the antagonistic relationship between Zn and Fe results in root chelation issues and competition for xylem cell entry [58]. The insufficient Fe and Zn uptakes after their relevant foliar applications in the present study contradict previous reports, including on rosemary plants [29] and dragonhead plants (*Dracocephalum moldavica* L.) [30], where Fe and Zn accumulation were increased following their foliar applications. It can be also concluded that the non-accumulation of Fe and Zn inside the plant tissue in the present study after the additional application of the corresponding elements might be due to the fact that the plants were cultivated hydroponically, so they had all the nutrients in sufficient levels available at the root system and there was no need for additional minerals to enter through the leaf, as they would not be used and could even cause additional stress [44,45].

**Figure 5:**
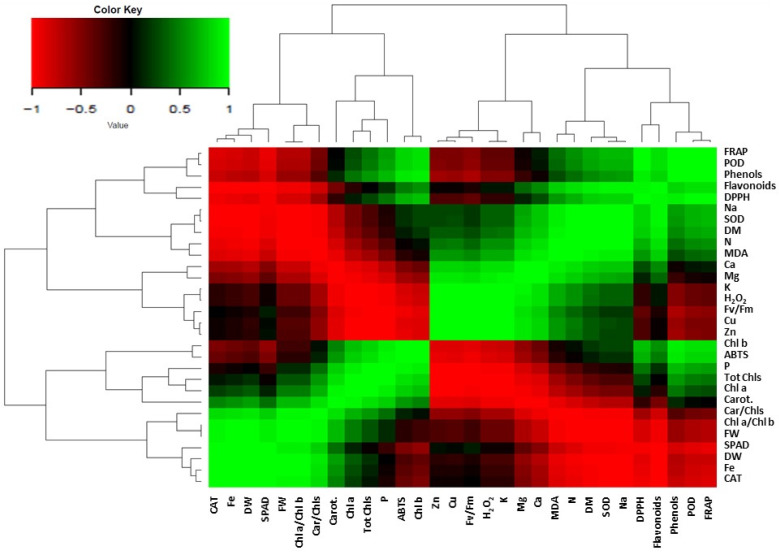
Heat-map matrices of the correlation between growth and physiological traits in *Sideritis cypria*. Each square indicates r (Pearson’s correlation coefficient of a pair of metabolites).

The correlation matrix of the plant growth and physiological traits is presented in Figure 5. Among the parameters that were activated, positive correlations were found for the increased fresh weight with the increased dry weight, Fe content, SPAD and CAT enzymatic activity. The above parameters were negatively correlated with the total phenols; flavonoids; antioxidant status; and N, Na and Ca content. Additionally, the Zn content in the plant tissue was positively correlated with the chlorophyll fluorescence and Cu, K, Mg and Ca content but negatively correlated with the P content.

**Figure 6:**
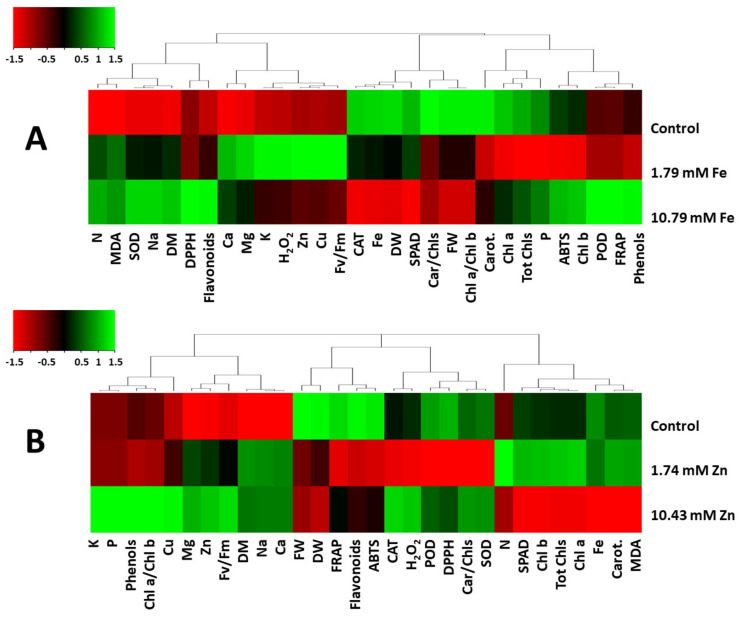
Metabolite changes in *Sideritis cypria*. Heat map representing relative expressions of growth and physiological traits elicited in plant tissue following foliar applications (**A**) with Fe and (**B**) with Zn as compared with control (water-sprayed) plants.

Heat maps (Figure 6) based on the relative expressions of plant growth, minerals and physiological traits of the *Sideritis* plants sprayed with Fe or Zn show a differentiation of plant responses among the treatments. The iron foliar application increased the N and Na content, with evidence of increased stress (MDA) and activation of enzymatic (SOD, POD) and non-enzymatic antioxidant mechanisms (phenols, flavonoids, antioxidant status) (Figure 6A). Noticeably, the mineral accumulation (Ca, Mg, K, Zn and Cu) in the plant tissue was increased only under the lower Fe levels (1.79 mM) of foliar application (Figure 6A). The zinc foliar application increased the mineral accumulation (K, P, Cu, Mg, Zn and Ca) but decreased the chlorophyll and Fe content (Figure 6B).

## 3. Materials and Methods

### 3.1. Experimental Setup, Plant Material and Growth Conditions

This experiment was conducted at the hydroponic infrastructures in a plastic multi-span greenhouse at Cyprus University of Technology, Limassol, Cyprus. The hydroponic system was based on the principles of the NFT (Nutrient Film Technique). Fifteen twin white plastic NFT channels (each measuring 4 m in length) were aligned with 15 catchments (60 L) to create 15 independent hydroponic units for the *S. cypria* cultivation. Each unit had a pair of plastic channels and was supported by a separate 60 L water replenishment tank. The nutrient solution (NS) absorbed by the plants was restored by automatically refilling with water from the replenishment tank. Every day, the electrical conductivity (EC) and pH were adjusted via the addition of the proper volumes of stock fertilizer solutions. Each hydroponic unit accompanied 12 plants, resulting in a final plant density of 25 plants/m^2^; a total of 180 plants were used. The average air temperature and humidity in the greenhouse were 24.9 °C and 52.7%, respectively, during daylight hours.

The plant seedlings were purchased from the Department of Aromatic Plants, Ministry of Agriculture, Cyprus. The seedlings were transplanted into netted pots filled with perlite and placed into the pot positions of the NFT channels and were kept for 20 days under a standard NS to allow recovery from the transplanting stress. Based on the previous results [59], the tested decreased-P (Ν150/P50/K350) nutrient solution was selected for the *S. cypria* in order to evaluate the effectiveness of the foliar applications with micronutrients. The nutrient concentration in the standard NS is presented in Table S1. After 20 days in the NFT system, the plants were subjected to the modified NS for 44 days (Table S1). All the NSs were checked daily and altered as necessary. The pH was lowered when necessary (since the water was alkaline) with 5% *v*/*v* H_2_SO_4_, and the EC was changed through the addition of the modified NS. The target pH and EC of the NS were 5.8 and 2.3 dS/m, respectively. The treatments were further split considering the application of (i) foliar spraying with pure dH_2_O (control), (ii) foliar dH_2_O with Fe (0.5 g/L FeSO_4_ · 7H_2_O or 1.79 mM Fe or 0.1 g/L Fe), (iii) foliar dH_2_O Fe (3.0 g/L FeSO_4_ · 7H_2_O or 10.79 mM Fe or 0.6 g/L Fe), (iv) foliar dH_2_O with Zn (0.5 g/L ZnSO_4_ · 7H_2_O or 1.74 mM Zn or 0.114 g/L Zn) and (v) foliar dH_2_O with Zn (3.0 g/L ZnSO_4_ · 7H_2_O or 10.43 mM Zn or 0.68 g/L Zn). The foliar applications took place four times at 10-day intervals. The concentration, as well as the spraying intervals, was based on preliminary findings [25] as well as previous reports on basil with 0.5 g/L Fe; 0.5 g/L Zn [60] or 0.41–0.61 g/L Zn [22] and on spearmint with 0.14 g/L Zn [53]. Spraying was implemented in the morning using a hand sprayer. During cultivation, no chemicals and pesticides were used.

### 3.2. Plant Growth Measurements

Following 44 days of the *S. cypria* growth, six individual plants—from different replicated units—for each treatment were considered for detailed plant growth analysis. Plant fresh upper biomass (g) and dry weight (g) were measured, and the dry matter content was computed as percentage values (%).

### 3.3. Plant Physiology and Photosynthesis-Related Measures

Leaf photochemistry features were also evaluated; relative chlorophyll content values were obtained with the use of an optical chlorophyll meter (SPAD-502, Minolta, Osaka, Japan). The leaf chlorophyll fluorescence of PSII (Fv/Fm) was measured with the OptiSci OS-30p Chlorophyll Fluorometer (Opti-Sciences, Hertfordshire, UK). Chlorophyll extraction was also performed using fresh plant tissue (six replications/treatment; each replication was a pool of two plant tissues; 0.1 g). Photosynthetic leaf pigment, chlorophyll a (Chl a), chlorophyll b (Chl b), total chlorophylls (t-Chl) and carotenoid content were then computed (mg/g fresh weight) [61,62]. The ratios of Chl a:Chl b and carotenoids:total Chls were computed too.

### 3.4. Plant Tissue and Nutrient Ion Concentration Analysis

At the end of the experiment, samples from the upper parts of the plant (leaves and stems) were used to measure the mineral content in six replications per treatment (three pooled plants per replication). Dry material was ash-burned at 450 °C and acid-digested (2 M HCl). The contents of potassium (K), sodium (Na) and phosphorous (P) were determined in accordance to Chrysargyris et al. [63], while the magnesium (Mg), calcium (Ca), copper (Cu), Fe, and Zn were by an atomic absorption spectrophotometer (PG Instruments AA500FG, Leicestershire, UK). The nitrogen was evaluated by the Kjeldahl method (BUCHI, Digest automat K-439 and Distillation Kjeldahl K-360, Flawil, Switzerland). The data were expressed in g/kg and mg/kg of dry weight (DW) for the macronutrients and micronutrients, respectively.

### 3.5. Total Phenols, Total Flavonoids and Antioxidant Activity

Six samples (two separate plants were merged for each sample) for each treatment were used for the polyphenol methanolic extraction, and the methanolic extracts were stored at −20 °C until analysis for the total phenolic and flavonoid content. The total antioxidant activity was assessed by using three tests: the 2,2-diphenyl-1-picrylhydrazyl (DPPH), ferric reducing antioxidant power (FRAP) and 2,2′-azino-bis(3-ethylbenzothiazoline-6-sulphonic acid (ABTS) methods.

The total phenolic content measurement was assessed with the Folin–Ciocalteu method (at 755 nm) [64]. The results were expressed in gallic acid equivalents (mg GA/g fresh weight, FW). The content of the total flavonoids was assessed using the aluminum chloride colorimetric method [65] as adjusted by Chrysargyris et al. [66]. The absorbance was measured at 510 nm. The total flavonoid content was expressed as rutin equivalents (mg rutin/g FW).

Free radical-scavenging activity was assessed as descripted previously [6]. Briefly, the DPPH radical-scavenging activity of the leaf extracts was measured at 517 nm while the FRAP activity was measured at 593 nm, as described by Chrysargyris et al. [6]. The ABTS assay was implemented according to the methodology described by Wojdylo et al. [67]. Results were expressed as Trolox ((±)-6-Hydroxy-2,5,7,8-tetramethylchromane-2-carboxylic acid) equivalents (mg Trolox/g FW).

### 3.6. Lipid Peroxidation, Hydrogen Peroxide and Enzyme Antioxidant Activity

The content of hydrogen peroxide (H_2_O_2_) was determined according to Loreto and Velikova [68]; six samples (two separate plants were merged per sample) for each treatment. The H_2_O_2_ concentration was measured with standards ranging from 5 to 1000 μM of H_2_O_2_, and the calibration curve was displayed appropriately. Both the samples and standards were measured at 390 nm, and the results were expressed as μmol H_2_O_2_/g FW.

Lipid peroxidation, in terms of malondialdeyde content (MDA), was assessed according to De Azevedo Neto et al. [69]. The absorbance was measured at 532 nm and corrected for non-specific absorbance at 600 nm. The MDA content was determined using the extinction coefficient of 155 mM/cm. The results were expressed as nmol of MDA/g FW.

The activity of the antioxidant enzymes for superoxide dismutase (SOD) (EC 1.15.1.1) and for catalase (CAT) (EC 1.11.1.6) were assayed as previously reported by Chrysargyris et al. [70], and the absorbance was determined at 560 nm for the SOD and at 240 nm for the CAT. The peroxidase activity (POD) (EC 1.11.1.6) was determined following the increase in absorbance at 430 nm, as previously reported by Chrysargyris et al. [71]. The results were expressed as enzyme units per mg of protein. The protein content in the leaf tissue was measured using the Bradford method and bovine serum albumin as a standard.

### 3.7. Statistical Methods

The data were analyzed by IBM SPSS v.22 for the analysis of variance (ANOVA), and the results are shown as means ± standard error (SE). Duncan’s multiple-range tests were conducted when the ANOVA had a significant treatment impact at *p* < 0.05. Pearson’s correlation test was used to determine the correlation coefficients between the foliar treatments of Zn and Fe, with respect to different parameters. The pairwise metabolite effect correlations were calculated by Pearson’s correlation test using the R program (R version 3.6.2; 12 December 2019).

## 4. Conclusions

The present study examined the effects of Fe and Zn foliar spraying on the growth parameters, physiology and nutrient content of *Sideritis cypria* plants grown in hydroponics. As an unexplored and endangered species, *S. cypria* is a MAP of great importance for the Mediterranean area, and after its successful introduction to hydroponic cultivation, the mineral needs and a potential biofortification plan to enrich the plant’s performance and properties are of great importance. This was the scope of this research, and the results appear quite promising in terms of mineral accumulation and antioxidant properties, besides the decrease in growth parameters.

The high Fe levels and both Zn levels (1.74 mM and 10.43 mM Zn) resulted in reduced plant fresh biomass and dry matter content. Additionally, the application of the high Fe levels (10.79 mM Fe) increased the contents of the total flavonoids and the non-enzymatic antioxidative mechanisms of the plants. On top of that, the high Fe levels resulted in increased oxidative stress, as was indicated by the high MDA and hydrogen peroxide content and the increased POD enzymatic activity. The foliar applications of Fe and Zn significantly affected the accumulation of minerals inside the plant tissue; the Na content was increased in all cases, while the Zn application stimulated the Ca content and the Mg levels were increased only in plants sprayed with the high Zn levels (10.43 mM Zn). None of the tested minerals (Zn or Fe) had any impact on the accumulation of Zn or Fe inside the plant tissue. Biofortification with minerals can enhance the nutritional value of MAPs and promote secondary metabolites. However, further research is needed to examine how biofortification affects the biocidal features of the produced plant extracts and/or EOs of plant species subjected to biofortification schemes.

## Figures and Tables

**Table 1:** Effect of foliar applications (foliar with dH_2_O—control, 1.79 mM Fe, 10.79 mM Fe, 1.74 mM Zn and 10.43 mM Zn) on *Sideritis cypria* upper fresh weight (FW; g/plant), dry weight (g/plant) and dry matter (DM; %) in plants in hydroponics.

Foliar Applic.	Biomass FW	Biomass DW	Biomass DM
**Control**	84.83 ± 5.22 a	9.80 ± 0.59	11.57 ± 0.24 b
**1.79 mM Fe**	68.13 ± 6.86 ab	8.83 ± 0.89	12.97 ± 0.15 ab
**10.79 mM Fe**	57.18 ± 9.48 b	7.81 ± 1.24	13.75 ± 1.09 a
**1.74 mM Zn**	60.00 ± 6.75 b	8.54 ± 0.97	14.21 ± 0.34 a
**10.43 mM Zn**	57.43 ± 3.67 b	8.04 ± 0.49	14.02 ± 0.57 a

Significant differences (*p* < 0.05) among foliar applications are represented by different letters.

**Table 2:** Effects of foliar applications (foliar with dH_2_O—control, 1.79 mM Fe, 10.79 mM Fe, 1.74 mM Zn and 10.43 mM Zn) on *Sideritis cypria* SPAD values, leaf chlorophyll fluorescence (Fv/Fm), chlorophyll content (a, b, total; mg/g fresh weight), carotenoid content (mg/g fresh weight) and Chl a:Chl b and carotenoids:total Chls ratios in plants grown in hydroponics, with NPK values of 150-50-350 mg/L.

Foliar Applic.	SPAD	Chlorophyll Fluorescence	Chlorophyll a	Chlorophyll b	Total Chlorophylls	Carotenoids	Chlorophyll a/Chlorophyll b	Carotenoids/Total Chlorophylls
**Control**	51.97 ± 2.11 ab	0.80 ± 0.004	1.05 ± 0.04 ab	0.38 ± 0.02	1.43 ± 0.06 ab	0.23 ± 0.010 a	2.82 ± 0.07	0.16 ± 0.000 a
**1.79 mM Fe**	50.55 ± 5.79 ab	0.82 ± 0.021	0.77 ± 0.03 d	0.32 ± 0.06	1.09 ± 0.08 d	0.14 ± 0.006 c	2.54 ± 0.40	0.13 ± 0.013 b
**10.79 mM Fe**	47.17 ± 2.16 ab	0.80 ± 0.025	0.95 ± 0.03 bc	0.41 ± 0.03	1.35 ± 0.04 bc	0.18 ± 0.012 b	2.37 ± 0.21	0.13 ± 0.007 b
**1.74 mM Zn**	55.28 ± 3.77 a	0.81 ± 0.007	1.15 ± 0.02 a	0.41 ± 0.02	1.56 ± 0.04 a	0.24 ± 0.003 a	2.79 ± 0.09	0.15 ± 0.000 ab
**10.43 mM Zn**	43.3 ± 2.30 b	0.82 ± 0.012	0.90 ± 0.05 c	0.30 ± 0.03	1.19 ± 0.08 cd	0.19 ± 0.010 b	3.02 ± 0.18	0.16 ± 0.009 a

Significant differences (*p* < 0.05) among foliar applications are represented by different letters.

## Data Availability

The authors declare data availability only upon request.

## Supplementary Materials

The following supporting information can be downloaded at: https://www.mdpi.com/article/10.3390/plants14060840/s1, Table S1: Electrical conductivity (EC) and nutrient concentrations in the nutrient solution (NS) supplied to *S. cypria* plants grown in a closed hydroponic system.
