# Supplementary material for: Iron and Zinc Foliar Spraying Affected Sideritis cypria Post. Growth, Mineral Content and Antioxidant Properties
Source: Plants (Basel). 2025 Mar 7;14(6):840. doi: 10.3390/plants14060840 (PMC11944779; doi:10.3390/plants14060840)
Supplement: Supplementary file 1 [file plants-14-00840-s001.zip › plants-3487601-supplementary.pdf]

Table S1. Electrical conductivity (EC) and nutrient concentrations in the nutrient solution (NS) supplied to *S. cypri*a plants grown in a closed hydroponic system. The pH of the nutrient solution was set at 5.8 using H_2SO_4 (5% v/v).

Parameters	Starter NS	Modified NS
EC dS m^{-1}	1.75	2.57
K^+ mM	7.55	8.95
Ca^{2+} mM	3.50	3.74
Mg^{2+} mM	1.00	2.88
NH_4^+ mM	0.5	0.62
NO_3^- mM	13.72	10.71
SO_4^{2-} mM	1.29	1.56
$H_2PO_4^-$ mM	1.80	1.63
Cl^- mM	1.49	1.49
Fe μM	30.00	71.56
Mn μM	5.00	18.21
Zn μM	4.00	1.53
Cu μM	1.00	4.72
B μM	30.00	18.52
Mo μM	0.50	0.52
K/Total N		0.79
K/(K+Ca+Mg)		0.40
Ca/(K+Ca+Mg)		0.34
Mg/(K+Ca+Mg)		0.26

Starter nutrient solution (NS) was introduced into the closed system for 20 days, before the application of the modified NS as 150 mg N/L, 50 mg P/L, and 350 mg K/L.
